# The emotional labour of academia in the time of a pandemic: A feminist reflection

**DOI:** 10.1177/1473325020981089

**Published:** 2021-03

**Authors:** Michelle Newcomb

**Affiliations:** Faculty of Health, School of Public Health and Social Work, Queensland University of Technology, Queensland, Australia

**Keywords:** Social work education, feminism, critical reflection, emotional labour

## Abstract

During the COVID19 pandemic, emotional labor has become an indispensable resource in social work, providing comfort, strength, and focus for many. Within the social work academy, emotional labor has been required to support students, especially as education has moved quickly into online and remote teaching modes. For the majority female social work educators, the pandemic has also led to a rise in caring responsibilities, especially for children. This personal essay explores the experience of a female, early career social work academic in negotiating the use of emotional labor simultaneously in paid and unpaid roles during the pandemic. This exploration is contextualised within the neoliberal university and its expectation of how emotional labor should be used to meet student and business needs. The essay questions the individualized practice and responsibility of emotional labor and questions alternative ways to meet the emotional needs of individuals, families, and universities during the COVID19 pandemic.

I watched the world succumb to COVID19, but nothing prepared me the emotional labour which would be expected of me. As a social work academic, I had intellectually anticipated emotions, disconnection and even death during the pandemic, but the lack of value prescribed to the emotional labour was unexpected. I work in a university in Australia and I am the primary carer of two children. Before the pandemic, I thought of myself as a high-performance juggler, throwing all my selves through a precarious, neoliberal academic existence. But during the pandemic I not only dropped balls I questioned their necessity. My ability to separate; the academic, the mother; the wife; the daughter and the friend vanished. Instead I juggled my own and other’s feelings of fear, anxiety, anger, and frustration without the required physical, mental, and emotional space. I felt a real and acute burden thrust upon me to maintain the emotions of others. This emotional labour extended to my children, the students enrolled in my class, but it was also used in maintaining the illusion of master juggler within my professional role. This reflective essay examines my experience of emotional labor in the time of COVID19 within the context of being a female, early career researcher within a neoliberal university. The essay provides a deep appreciation of how emotional labour is undervalued within academia; despite the vital role it has played globally in surviving the pandemic.

## What is emotional labour?

The idea of emotional labour is often related to caring acts, roles, and emotions within paid and unpaid spheres (Hochschild, 2012). Within the world of paid employment, emotional labor is considered a professional skill, involving the suppression of private feelings in replace of more work-related or socially acceptable emotions (Hochschild, 2012; Mastracci, 2012). Emotional labor can be bought and sold, encompassing a part of a worker’s wage (Mastracci, 2012). The use of emotional labor is seen as vital for many frontline positions such as teaching, social work and health care, which are concurrently female dominated professions. For women emotions may be considered an important resource making relational work a source of income (Hochschild, 2012). However, women should only express the emotions associated with their professional role, such as empathy otherwise they may be they may risk being considered less competent, irrational and lacking in leadership qualities (Rogers, 2017).

The term emotional labor has been explored and examined extensively by feminists (Hartley, 2018). Unpaid, feminised work such as child rearing or caring despite its lack of paid or social recognition also requires extensive emotional labor. For women engaged in both unpaid and paid work, the impacts of emotional labor can lead to feelings of fatigue, frustration and being overwhelmed (Hartley, 2018). In the time of COVID-19 pandemic I was able to examine my own experience of emotional labor as I struggled with managing my own and other’s feelings of uncertainty.

## The reflective process

Within this essay I have drawn from a critically reflective process to examine the messy and emotional experience of being a female academic during COVID19. In writing this, I do not proclaim to speak a universal or even generalisable truth rather I am exploring my own unique perspectives and identities alongside my perceptions and emotions during the pandemic. I have used a range of data in this article, including a personal journal, emails and recollections of conversations. I have engaged in critical reflection, which allows me to analyse unjust power relations. Drawing from the work of Fook and Gardner (2007) I have used both critical theory and post structuralism to examine hegemonic, neoliberal discourse within higher education. I have taken care to de-identify individuals throughout the process of writing this and do not seek to blame or vilify individuals or institutions. This essay provides a timely, accessible account of this unique period from my lived experience and adds to existing contributions about the role of women and emotional labor in academia (Broadbent et al., 2017; Laing and Laing, 2011; Lipton, 2017; May, 2011).

## Emotional labor and the neoliberal university in the time of COVID19

Universities have traditionally been designed and developed by and for men (Rogers, 2017). This means female academics can often face disadvantages in terms of career progression or a lack of status being afforded to feminist areas of interest (Broadbent et al., 2017; Lawless, 2018; Lipton, 2017). For female academics who are also parents, tension can be felt in meeting the physical, temporal, and emotional needs of both students, colleagues and their own children. Within the neoliberal university a division of labor between pastoral or caring roles is often seen between men and women, with women taking on the majority of this work (Gajparia, 2017; Lawless, 2018). For myself, the emotional load of supporting students in the transition to online learning seemed to blur at times with students’ image of me as a woman and mother. I journaled:

*“Students are so stressed with changes and technology; they want me to soothe and comfort them. Many are losing jobs, soon they might be losing family members. I do not know how to support them … Their anxiety about the world is transmitted to me.”*

Although guidance had been provided to students from myself, the faculty and the university, students wanted additional reassurance. Emails from students suggested how they were *“not good with technology”* required comforting and fortifying messages of support. However, these messages of support and comfort were also required by my own family. My own emotions which included fear and reticence about remote, emergency online teaching seemed to have no space for expression. I felt that as a female academic my ability to engage in emotional labor was both expected but also invisible (Lawless, 2018).

My own wellbeing began to suffer, because the needs of colleagues, students and my own family had to be prioritised. Initially I was required to continue lecturing and tutoring students to the same timetable despite my children now being home-schooled. I wrote in my journal:

*“I found myself teaching small children, trying to find routine, patience and resources. Their needs soaked into my working day with learning activities, arguments and boredom all needing to be attended to, whilst working. My children have become anxious about disease, infection and isolation. My seven-year-old insisted she could only play in my bed, where she built pillow forts for an entire week.”*

Whilst individual managers and staff acknowledged the difficulties working academic mothers faced my role as a parent remained largely invisible. I felt deeply uncomfortable with my children being part of meetings, lectures, and tutorials, where they became at best a cute distraction and at worst a symbol of my reduced capacity to meet workplace expectations. During the pandemic I carried many roles, mother, partner, daughter, lecturer, researcher, and confidant often literally within the same chair. These roles were varied and constantly changing requiring a great deal of concentration and often immediate responses.

Balancing multiple roles within the domestic sphere was coupled with the increasing workload as my institution moved to a virtual campus. Pressure to keep students engaged and enrolled was felt and I found myself working day and night, weekends and early mornings transferring and creating content into an online format. Simultaneously, I was bombarded with emails about how I should now be teaching, new approval processes and innovative ideas for online assessment. These emails and suggestions seemed to add to my ever-growing list of tasks and found myself overwhelmed with the volume of work. Childless colleagues seemed more able to focus and concentrate and I became aware that motherhood might be a penalty in my success as an academic.

Initially the university offered ideas on how to survive the pandemic including advice on time management or working from home with children. However, these suggestions added to my “to-do” list and created a sense of failure within me. It felt that these suggestions were simply mechanisms to imply the university was engaging in the practice of care, without providing practical support (Rogers, 2017). The suggestions raised questions of paternalism rather than genuine care, where the institution knows better than the individual (Rogers, 2017). The social and professional expectations to perform well in all these roles became overwhelming.

Asked how I was in a team meeting one day I found myself in tears. For the first time in a long time someone had shown concern for me as a person. However, my emotional outburst made me feel embarrassed and ashamed. In displaying my tears, I had dropped my metaphorical juggling balls and revealed my true self. After my outburst, my supervisors checked upon me via email offering free external counselling. I worried that my emotions might make me seem unprofessional as emotional thought and behaviour within academia is often considered as somehow separate to the rational processes needed in the process of research and teaching (Rogers, 2017). I was perplexed as pandemic conditions meant emotion had become a prized component in both my paid and unpaid roles. Suddenly the institution was in my home every day, witnessing my family life. The university required me to use my emotions in a process of deep acting; I needed to placate, reassure and maintain composure with both students and staff however I could not authentically share my actual experience of the pandemic (Hochschild, 2012). Whilst the display and expression of empathy, warmth and understanding was an expected part of my role, my display of stress and displeasure was surprising to some. In crying I had transgressed the institution’s expectation of what a ‘good’ academic should be. Through a process of personal and collective reflection and resistance I was however able to consider new ways to frame my experience.

## Self-compassion and resistance

The cumulated emotion that lead to my teary breakdown, did not go unnoticed by my wider team. Kindness became apparent from colleagues who offered to conduct online classes or started to acknowledge the difficulties faced by parents. Emails began with the other parents on the team, concerns were shared, collated, and given to managers. The feeling of collectively struggling decreased my sense of hopelessness. It reminded me of the temporal nature of a pandemic and decreased my isolation.

Perhaps my greatest epiphany came from understanding how I had co-opted and accepted the idea of personal responsibility into my life. I had fallen into a neoliberal trap of assuming it was my role as an individual to limit or mitigate organisational risks (Green, 2007). In being a high-performance juggler, I was co-opting a neoliberal discourse which ran against my core beliefs. Whilst I was responsible for mitigating my family’s risk of contracting COVID19, I could not shoulder the universities risks in relation to student engagement and enrolments. I realised I could not be solely responsible for my children’s educational outcomes. I was not responsible for my students’ emotional well-being, or comfort with new technologies and ways of learning. In personalising this discourse of responsibility, I had become isolated and miserable.

I began to engage in a process of self-compassion based on restraint. No longer was I undertaking as much online, face to face teaching, instead I combined classes. As a designated ‘essential worker’ in Queensland, Australia I took up the opportunity to send my children to school. If we became sick, I would willingly take sick leave, supported by a well-resourced, universal health care system. I minimised the creation of additional teaching materials, using existing resources. I prioritised writing and research as a mechanism for survival and resistance to the individualised discourse of responsibility given to me (Green, 2007). Instead of focusing on what was wrong with the situation, I focused on how I could use this time to reject masculinist discourses about emotional labour and academia including writing this article.

Through this process of reflection, I found space for resistance. I’ve come to recognise emotional labour as a limited resource that needs greater acknowledgement (Lawless, 2018). Rather than expending this precious resource in maintaining neoliberalism, this experience has taught me to direct this resource where it matters; to myself and my family. It seems crude to commodify emotion for capitalism instead the pandemic as taught me to keep it for real, authentic connection.
